# Microbial dysbiosis index for assessing colitis status in mouse models: A systematic review and meta-analysis

**DOI:** 10.1016/j.isci.2023.108657

**Published:** 2023-12-06

**Authors:** Min-Ji Kim, Da-Ryung Jung, Ji-Min Lee, Ikwhan Kim, HyunWoo Son, Eun Soo Kim, Jae-Ho Shin

**Affiliations:** 1 Department of Applied Biosciences, Kyungpook National University, Daegu 41566, Republic of Korea; 2 Cell & Matrix Research Institute, Kyungpook National University, Daegu 41940, Republic of Korea; 3 NGS Core Facility, Kyungpook National University, Daegu 41566, Republic of Korea; 4 Division of Gastroenterology and Hepatology, Department of Internal Medicine, School of Medicine, Kyungpook National University, Daegu 41944, Republic of Korea

**Keywords:** Physiology, Microbiology, Microbiome

## Abstract

Although countless gut microbiome studies on colitis using mouse models have been carried out, experiments with small sample sizes have encountered reproducibility limitations because of batch effects and statistical errors. In this study, dextran-sodium-sulfate-induced microbial dysbiosis index (DiMDI) was introduced as a reliable dysbiosis index that can be used to assess the state of microbial dysbiosis in DSS-induced mouse models. Meta-analysis of 189 datasets from 11 independent studies was performed to construct the DiMDI. Microbial dysbiosis biomarkers, *Muribaculaceae*, *Alistipes*, *Turicibacter*, and *Bacteroides*, were selected through four different feature selection methods and used to construct the DiMDI. This index demonstrated a high accuracy of 82.3% and showed strong robustness (88.9%) in the independent cohort. Therefore, DiMDI may be used as a standard for assessing microbial imbalance in DSS-induced mouse models and may contribute to the development of reliable colitis microbiome studies in mouse experiments.

## Highlights

• Mouse model studies face limits in reproducibility due to small sample sizes
• A total of four genera were selected to construct the dysbiosis index for mouse model
• DiMDI demonstrated a high accuracy of 82.3% and showed strong robustness (88.9%)
• DiMDI is useful to evaluate microbial imbalance in DSS-induced mouse models

## Graphical abstract

**Figure:**
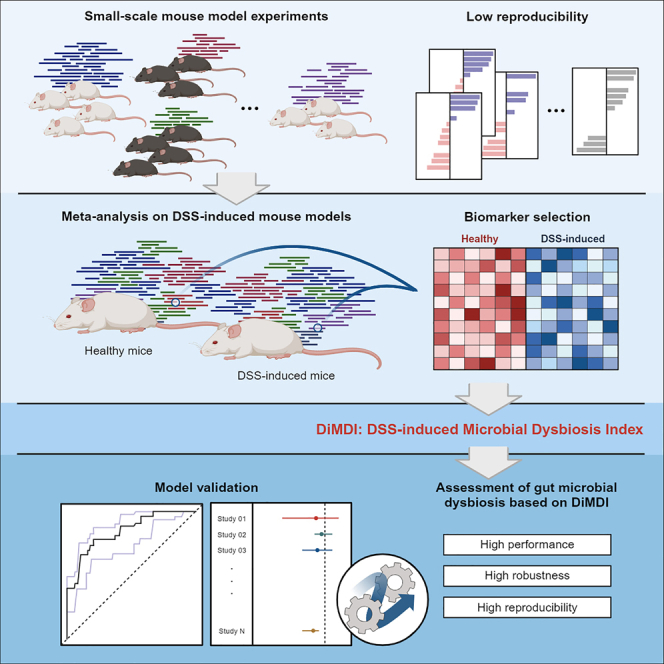

## Introduction

Over the last 20 years, research advances in human gut microbiome have revealed important connections and potential mechanistic insights into various complex chronic diseases, including metabolic syndrome, cancer, and autoimmune diseases.^1^^,^^2^ Microbiome studies have focused on elucidating the underlying pathophysiological mechanisms of diseases and devising potential intervention strategies. As numerous studies discover more about gut symbionts that play important roles in host health and disease, more studies have developed analytical tests or quantitative methods that provide health indicators based on the gut microbiome.^3^^,^^4^^,^^5^ Diseases can be diagnosed on the basis of the gut microbiome by utilizing polymerase chain reaction techniques.^6^^,^^7^ Thus, the fecal microbiome can be used as a faster and easier non-invasive method than traditional clinical diagnostic methods using blood or biopsy for predicting and diagnosing diseases. Furthermore, the gut microbiome is associated with not only gastrointestinal disorders but also the brain and skin diseases: therefore, an array of disease prediction and diagnostic models using the fecal microbiome is being developed.^8^^,^^9^^,^^10^

Inflammatory bowel disease (IBD), a major disease in gut microbial studies, is a gastrointestinal disorder characterized by acute and chronic inflammation. It includes conditions such as Crohn disease (CD) and ulcerative colitis (UC), whose etiologies have not been fully elucidated.^11^ In recent years, IBD has evolved into a chronic disease with a global impact. The clinical manifestations of IBD include primarily rectal bleeding, diarrhea, and tenesmus and occasionally lower abdominal pain.^12^ In the past few decades, several animal models have been developed to explore the molecular mechanisms of IBD and assess potential therapeutic interventions.^13^ Among animal models, the dextran-sulfate-sodium (DSS)-induced colitis model has gained significant popularity in IBD research because of its rapidity, simplicity, and controllability.^14^ DSS, a water-soluble negatively charged sulfated polysaccharide with anticoagulant properties, can potentially induce an inflammatory pattern in the mouse intestinal tract that is similar to human UC; however, the exact mechanism remains unknown.^15^ Moreover, acute, chronic, and recurrent models of intestinal inflammation can be easily established by adjusting the concentration and frequency of DSS administration. Because of the DSS-induced damage to the intestinal lining of mice, their intestinal environment and microbial community rapidly change. Given these advantages, numerous studies have examined the gut microbial community associated with IBD by using DSS-induced mouse models.

Typically, studies involving mouse models use sample sizes ranging from 3 to 10 for each group. However, such studies with limited sample sizes may have a reduced result reliability because of potential statistical analysis errors and subject bias.^16^ Moreover, establishing a consistent baseline becomes challenging because such studies are performed in various laboratories. In microbiome studies, results can vary based on biases in DNA extraction, sequencing, bioinformatics analysis methods, and experimental conditions.^17^^,^^18^ In line with the active pursuit of biomarker discovery and index construction in human studies, a consistent microbial imbalance model in mouse studies should be developed. Several previous studies have attempted to develop dysbiosis index in mouse studies, but they were conducted in individual small-scale studies and lacked validation that the models could be representative of dysbiosis in the mouse model.^8^ Thus, the development of microbial imbalance models through a comprehensive analysis will secure a reliable disease-associated microbial community for therapeutic research.

To ensure high reproducibility in mouse experiments with small sample sizes, we comprehensively analyzed microbiota studies involving DSS-induced mouse models. We introduced the “DSS-induced microbial dysbiosis index (DiMDI),” a reliable dysbiosis index that can be used to assess the state of microbial dysbiosis in DSS-induced mouse models. Microbial biomarkers, namely, *Muribaculaceae*, *Alistipes*, *Turicibacter*, and *Bacteroides*, were selected through four different biomarker selection methods and used to construct the DiMDI. Our established dysbiosis index had a high accuracy of 82.3% and showed strong robustness in the independent cohort. With its high accuracy, applicability across experimental conditions, and usefulness in intervention studies, the DiMDI serves as a standard for microbial imbalance in mouse models and holds the potential for advancing the understanding of complex interactions between the microbiota and disease outcomes.

## Results

### Meta-dataset of integrated mouse fecal microbiome data

**Figure 1:**
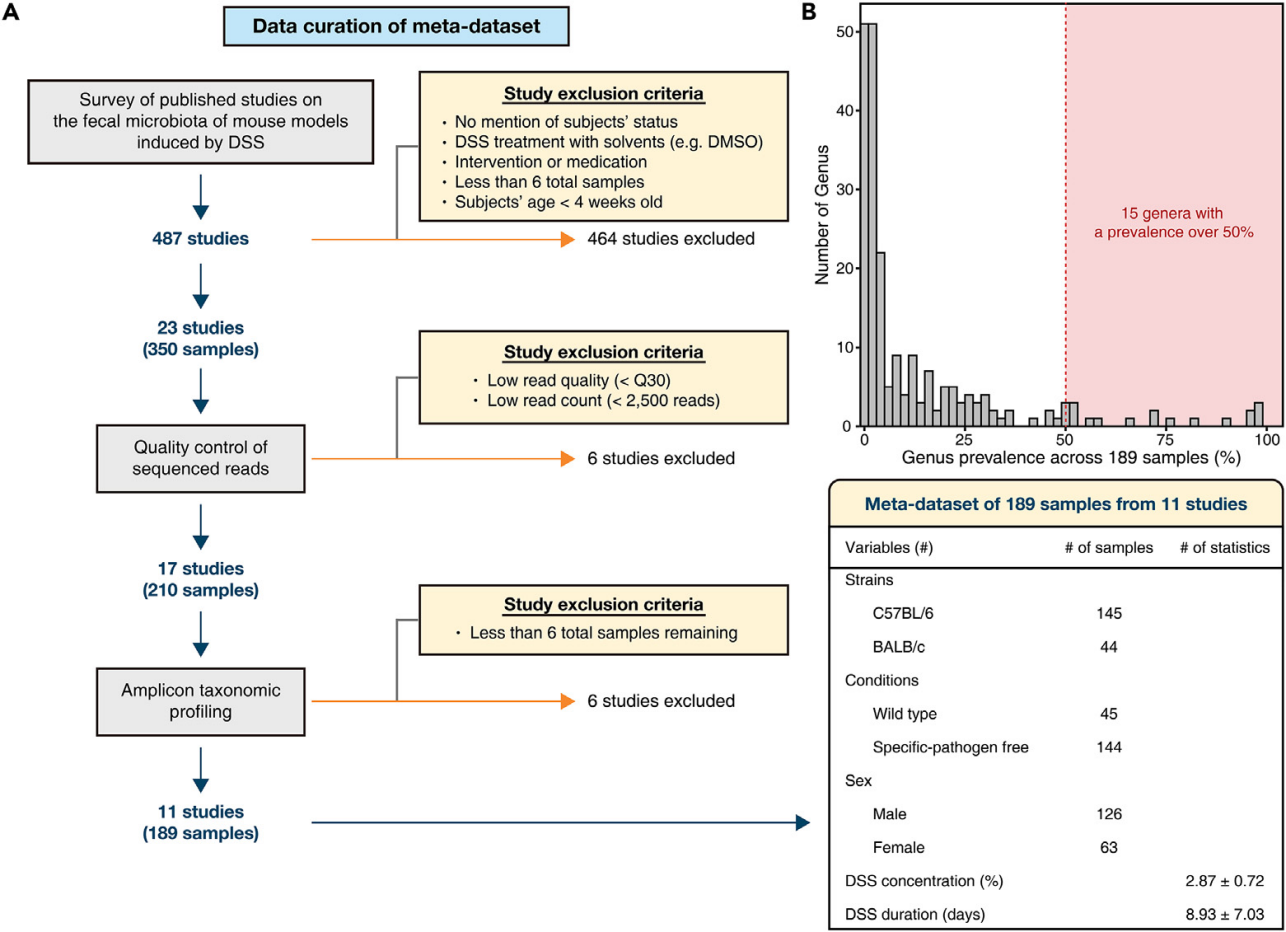
Workflow of meta-dataset compilation (A) Schematic illustration of the curation of amplicon sequencing meta-dataset. Initially, published studies on the fecal microbiota in DSS-induced mouse models were obtained from PubMed and Google Scholar. The amplicon data were retrieved from the NCBI Sequence Read Archive (SRA) and subsequently subjected to uniform reprocessing via a standardized bioinformatic method. Within the dataset, studies with low sequence quality, samples with a low number of reads remaining after quality control (QC), or studies with a small number of samples were excluded from further analysis. As a result, a total of 189 samples from 11 studies met the quality criteria and were retained for subsequent analysis. (B) Distribution of the prevalence of microbial genera across the 189 mouse fecal samples. After chloroplast, mitochondrial, and unknown/unclassified ASVs were removed, a total of 213 genera were retained. Among them, the rarely observed genera (i.e., detected <50% of all samples; 196 genera) were further excluded for discovering DSS-induced colitis microbial biomarkers.

An overview of meta-dataset compilation is depicted in Figure 1A. Healthy control (HC) samples were defined as those that were reported as no overt disease or any intervention at the time of the original study. Alternatively, unhealthy (UH) samples were defined as those that were treated with DSS. Accordingly, 350 mouse fecal microbiota from 23 studies were compiled. The number of the observed features was evaluated according to the read counts by using rarefaction curves (Figure S1). A total of 117 samples were excluded with having a read count below 2,500, a minimum read count at which the observed feature number converged. Moreover, 12 studies with low read quality, read count, and small samples after quality control of the sequences were excluded for further analysis. Finally, a total of 189 mouse fecal microbiota from 11 studies were used to construct the DiMDI. After raw amplicons were processed, genus-level classification profiling was performed; thus, 213 genera were detected. After rarely observed, unknown/unclassified genera were removed, 15 genera were left for further analysis (Figure 1B). Notably, the cutoff to determine the rarely observed genera was set by 50% of prevalence to increase the reproducibility in multiple independent studies.

In the present study, DiMDI was established to identify microbial structures in colitis-associated dysbiosis across various confounders such as mouse strain, sex, and conditions by obtaining information on mouse strains and conditions from mouse model studies. This information did not show significant differences between HC and UH groups (Table 1). Thus, all subsequent statistical analyses were performed without considering the individual conditions.

**Table 1:** Sample information of the study subjects

	HC (n = 98)	UH (n = 91)	p value^a^
Strains (n, %)			1
C57BL/6	75 (76.53)	70 (76.92)	
BALB/c	23 (23.47)	21 (23.08)	
Conditions (n, %)			0.955
Wild type	24 (24.49)	21 (23.08)	
Specific pathogen-free	74 (75.51)	70 (76.92)	
Age (weeks)	7.833 ± 1.618	8.296 ± 1.664	0.077
Sex (n, %)			0.959
Male	66 (67.35)	60 (65.93)	
Female	32 (32.65)	31 (34.07)	
DSS concentration (%)	n.a.	2.868 ± 0.722	<0.001
Treatment duration (days)	n.a.	8.934 ± 7.033	<0.001

a t test and chi-squared test were performed on continuous and categorical variables, respectively.

### Distinct microbiome in feces from mice with and without DSS induction

**Figure 2:**
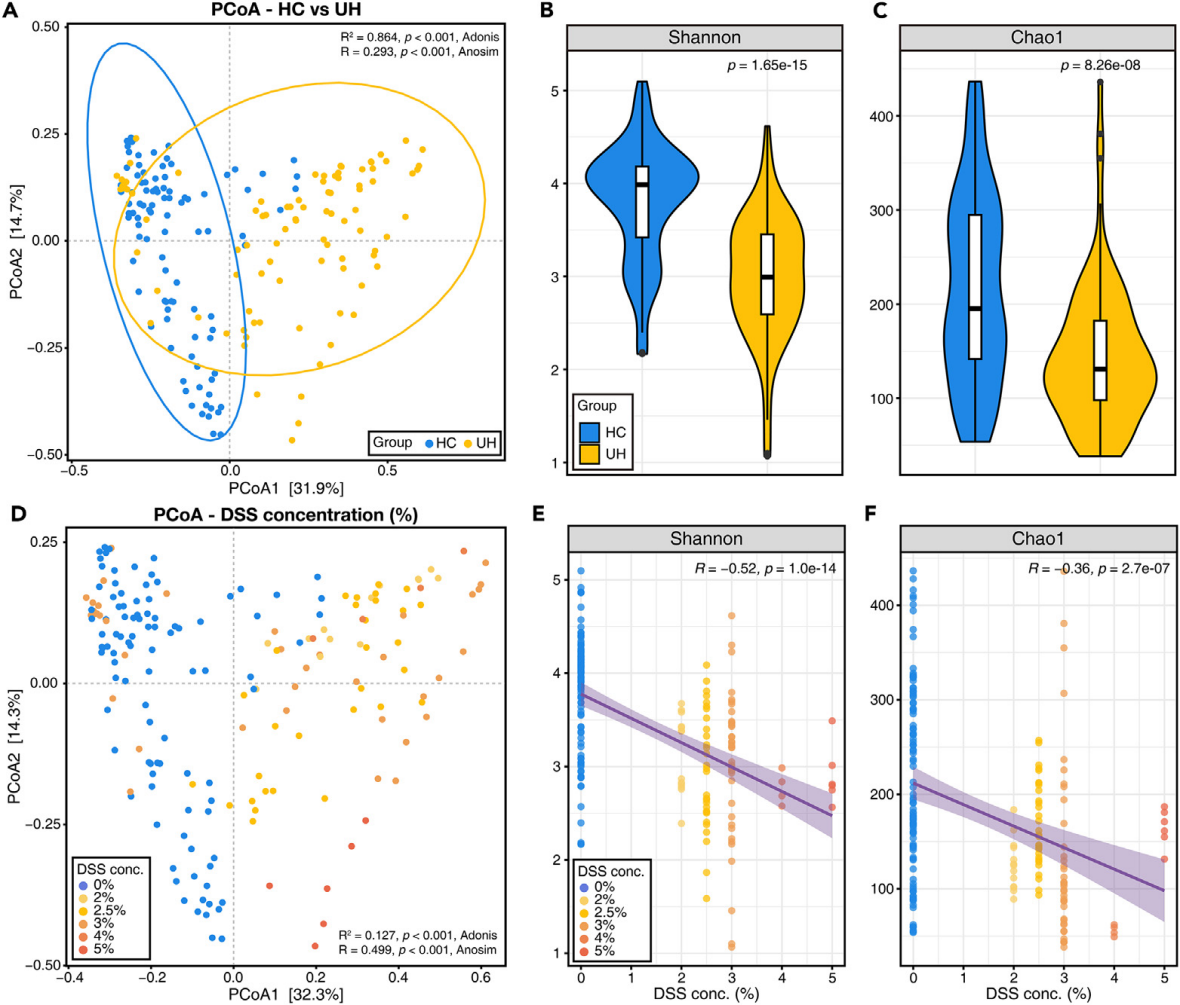
Distinct fecal microbial structures between healthy and colitis mouse models (A) Beta diversity (principal coordinate analysis; PCoA), (B) Shannon, and (C) Chao1 indices of mouse fecal microbiome according to healthy (HC) and DSS-induced colitis (UH) groups. t test was performed to compare the alpha diversity between HC and UH groups. (D) PCoA, (E) Shannon, and (F) Chao1 indices of the mouse fecal microbiome according to the concentration of DSS induction. Pearson correlation coefficient was used in correlation analysis.

The differences in the fecal microbiome between with (UH) and without DSS induction (HC) were analyzed using beta diversity and alpha diversity measures. For beta diversity, Bray–Curtis dissimilarities were calculated based on the amplicon sequence variants collapsed to the genus level. First, principal coordinate analysis (PCoA) revealed significant differences not only in dissimilarities (ANOSIM, R = 0.293, p = 0.001) but also in the distributions of fecal microbiota between the HC and UH groups (PERMANOVA, R^2^ = 0.864, p = 0.001; Figure 2A). When the concentration of DSS induction was also considered in the same PCoA plot, the dissimilarity and distribution according to DSS concentration were also significantly different (ANOSIM, R = 0.499, p = 0.001; PERMANOVA, R^2^ = 0.127, p = 0.001; Figure 2D). Alpha diversity showed that microbial diversity and species richness in mouse feces decreased in the UH group (Figures 2B and 2C). Consistently, the concentration of DSS induction negatively correlated with alpha-diversity indices (Figures 2E and 2F). These results indicated that DSS treatment sufficiently induced changes in the gut microbial community in the mouse model.

### Construction of DiMDI for identifying microbial dysbiosis in the mouse model

**Figure 3:**
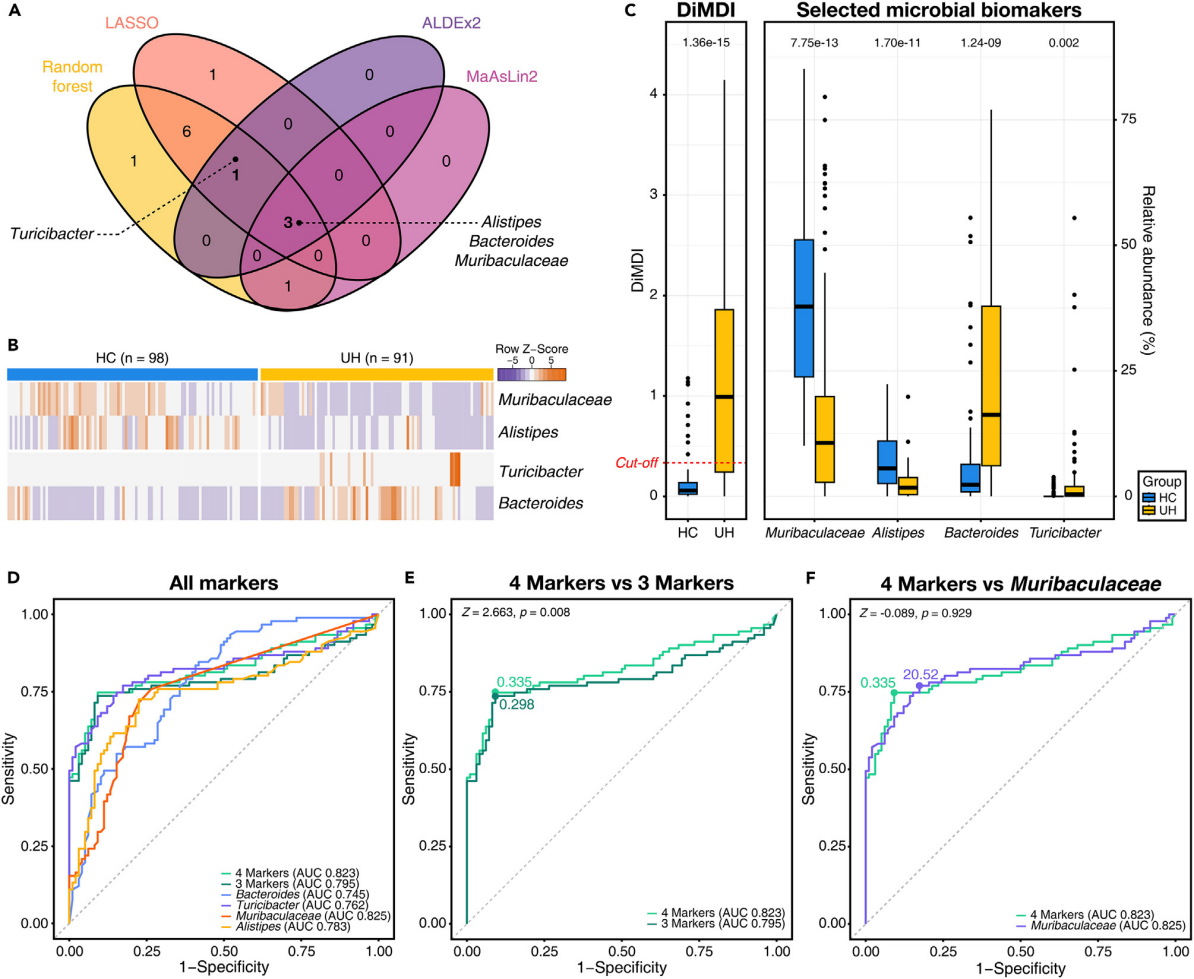
Construction of DiMDI using selected microbial biomarkers (A) Venn diagram showing the genera identified by four different feature selection methods. (B) Heatmap illustrating the relative abundance of the selected microbial biomarkers across all samples. (C) The calculated DiMDI and the relative abundance of the selected genera. The numbers within the figure represent p values obtained from the t test. (D) Area under the curve (AUC)-receiver operating curve (ROC) for DiMDI and each individual genus for classifying UH from HC. (E) Comparison of AUCs for DiMDI using either four or three markers. (F) Comparison of AUCs for DiMDI with four markers and the relative abundance of *Muribaculaceae*. DeLong test was performed to compare AUCs.

To determine microbial dysbiosis in the UH group compared with the HC group, we employed four different feature selection methods: random forest, L1-LASSO logistic regression, ALDEx2, and MaAsLin2. Random forest and L1-LASSO logistic regression methods identified 12 and 11 genera as the most important features, respectively (Table S1). Conversely, ALDEx2 and MaAsLin2 identified four genera as the most important genera (Table S1). A total of four genera (*Alistipes*, *Bacteroides*, *Muribaculaceae*, and *Turicibacter*) were selected from more than three methods (Figure 3A). Among them, three genera except *Turicibacter* were selected from all four methods. The four selected genera had high values in both MeanDecreaseAccuracy and MeanDecreaseGini in the random forest model and were identified as features with high importance in random forest modeling (Figure S2). These four genera also had over 95% of effect sizes in the L1-LASSO logistic model (Figure S3). We further explored the distributions of potential selected biomarkers (Figure 3B). *Muribaculaceae* and *Alistipes* had a high relative abundance in the HC group, and *Turicibacter* and *Bacteroides* showed a higher relative abundance in the UH group than in the HC group. The four selected genera showed strong significant differences in the HC and UH groups (Figure 3C).

The DiMDI was established with two versions of the DiMDI by using the relative abundances of the selected biomarkers: one using four selected markers (*Muribaculaceae*, *Alistipes*, *Bacteroides*, and *Turicibacter*) and another using three selected markers (*Muribaculaceae*, *Alistipes*, and *Bacteroides*). The discriminative performance of these models was assessed using the ROC curves and compared against the relative abundance of each selected marker. The area under the ROC curve (AUC) was the highest for the relative abundance of *Muribaculaceae* (AUC 0.825), followed by the DiMDI model using four markers (AUC 0.823; Figure 3D). The performance significantly differed between the DiMDI models with four and three markers (Z = 2.663, p = 0.008; Figure 3E). However, no significant difference was found between the model solely on *Muribaculaceae* and DiMDI with four markers (Z = −0.089, p = 0.929; Figure 3F). The optimal cutoff value for distinguishing UH from HC using DiMDI with four markers was determined to be 0.335. With this cutoff value, the DiMDI values were significantly higher in the UH group than in the HC group (Figure 3C). Thus, the DiMDI was finally constructed using the four markers to identify microbial dysbiosis in the DSS-induced mouse model.

### Comparison of performances between DiMDI and other microbial indicators

**Figure 4:**
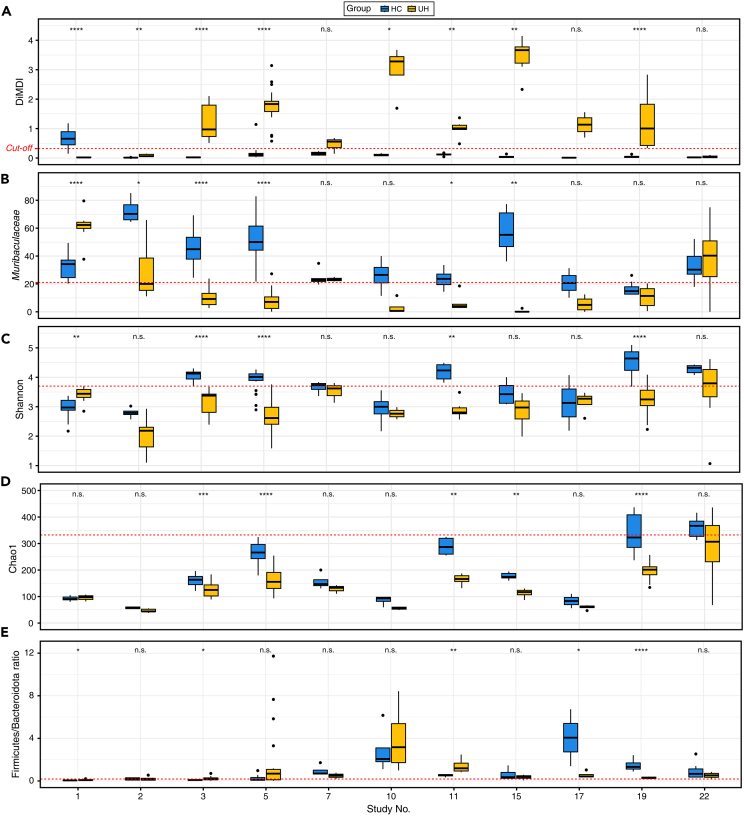
Comparative performances of DiMDI and other microbial indicators across study-specific scenarios The boxplot depicts (A) DiMDI, (B) the relative abundance of *Muribaculaceae*, (C) Shannon, (D) Chao1 index, and (E) the Firmicutes/Bacteroidota ratio across the two groups and studies. Wilcoxon signed-rank test was conducted to compare the two groups in each study. Significances were shown by n.s. (>0.05), ∗(<0.05), ∗∗(<0.01), ∗∗∗(<0.001), and ∗∗∗∗(<0.0001).

We investigated the differentiation ability of DiMDI and other microbial indicators (e.g., Shannon, Chao1 indices, and the Firmicutes/Bacteroidota ratio) between HC and UH within individual studies. The differentiation ability of the relative abundance of *Muribaculaceae* was also evaluated because it showed the highest performance in the ROC curve. By evaluating the individual study datasets, this approach not only mitigates a major source of batch variations but also offers an effective strategy to validate the consistency of our previously observed trends (comparing the HC and UH samples in the integrated groups) across multiple smaller-scale studies. The DiMDI in the HC group was significantly lower than that in the UH group in 7 out of 11 studies (Figure 4A). Although the DiMDI values were not statistically significant, these values in the two other studies (study 7 and 17) also distinguished two groups based on the cutoff. For the relative abundance of *Muribaculaceae*, Shannon diversity, and Chao1 index, we found significantly higher values in the HC group than in the UH group in four studies (Figures 4B–4D). In six studies, the HC and UH groups were not distinguished by the Firmicutes/Bacteroidota ratio, and three studies (studies 1, 3, and 11) showed significantly higher Firmicutes/Bacteroidota ratios in the UH group (Figure 4E). The identification performances of microbial indicators were also evaluated using ROC curves. The Firmicutes/Bacteroidota ratio showed the lowest performance with an AUC of 0.544 (Figure S4A). Chao1 index had a lower performance than the Shannon index (Chao1, AUC 0.711; Shannon, AUC 0.823). Although the Shannon index showed the same AUC value as the DiMDI (AUC 0.823; Figure S4B), the differentiation ability of DiMDI was higher than that of the Shannon index within individual studies. These results suggested that our established DiMDI generally outperformed other microbial indicators in distinguishing cases and controls.

### Validation of DiMDI reproducibility on independent study

**Figure 5:**
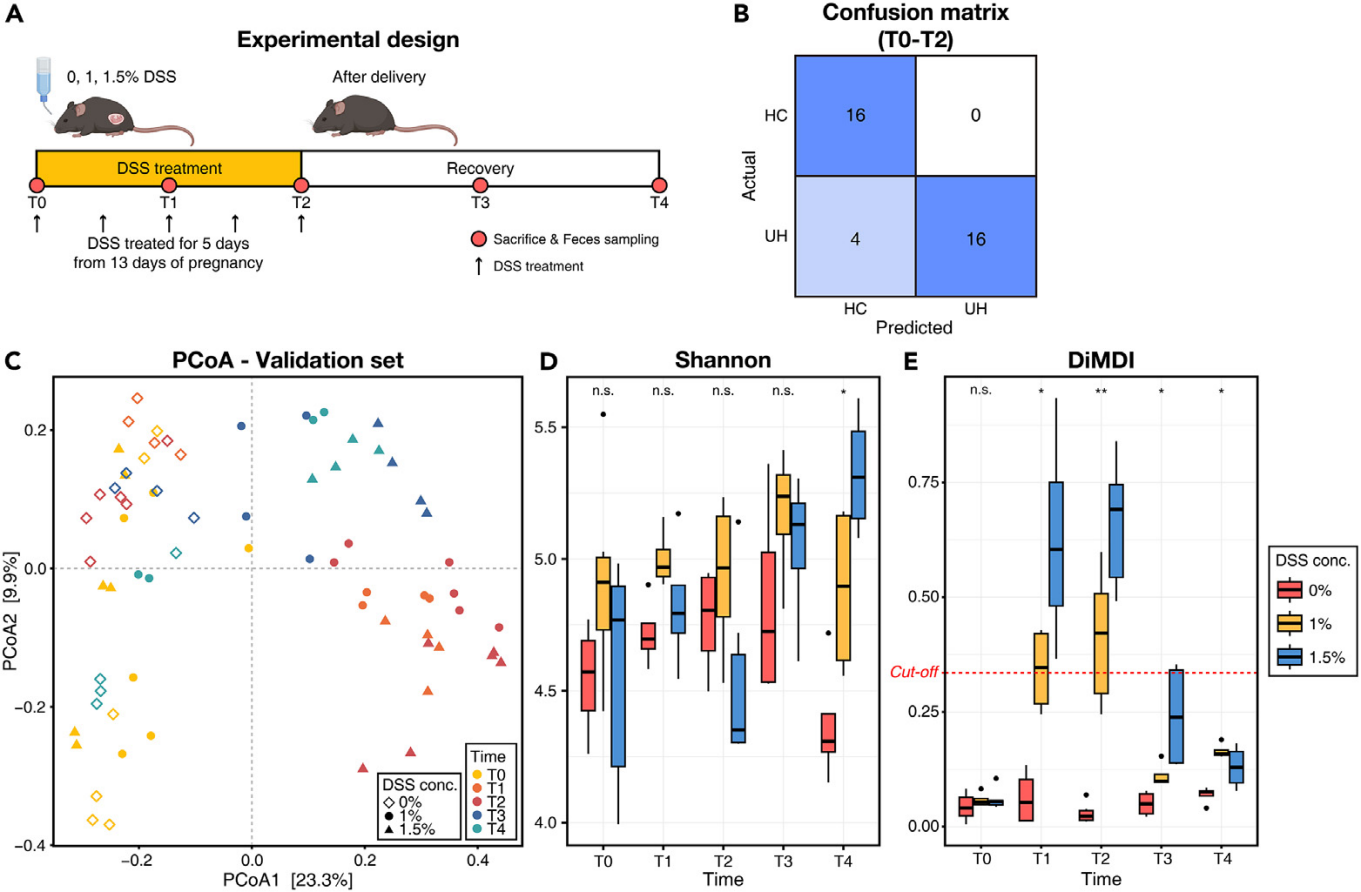
Validation of DiMDI on an independent study (A) Schematic of the experimental design. (B) Confusion matrix of the DiMDI, based on a cutoff of 0.335, applied to subjects observed from T0 to T2. (C) Principal coordinate analysis (PCoA) depicting variation in mouse fecal microbiota across time points and DSS concentrations. (D) Shannon index and (E) calculated DiMDI across time points and DSS concentrations. A Kruskal–Wallis test was used to compare three groups at each time point. Significances were shown by n.s. (>0.05), ∗(<0.05), and ∗∗(<0.01).

To evaluate the reproducibility of DiMDI, we applied the fecal microbiome data from the independent study and monitored their DiMDI values across the duration of DSS induction and recovery periods. Upon assessing the confusion matrix by computing DiMDI values throughout the DSS treatment period (from T0 to T2), we found that 4 out of 20 DSS-treated samples had DiMDI values below the established cutoff of 0.335 (88.9% accuracy; Figure 5B). The four samples with out-of-range DiMDI values were T1 and T2 time points of two subjects that were treated with 1% DSS. In addition, the confusion matrix for T0 and T1 predicted two samples in the UH group as HC group (Figure S5A). Otherwise, in T2 and T3, two false-positive samples and 1 false-negative sample were identified (Figure S5B). The PCoA plot showed that the microbial community structure did not revert to the baseline even during the two-week recovery period post-DSS treatment (Figure 5C). Moreover, the microbial communities immediately following five days of DSS treatment (T2) were found to be the most divergent from the baseline. The same trend was observed in DiMDI, which exhibited higher values in T2 with dose-dependent changes, decreasing during the recovery period toward a healthy microbial community (Figure 5E). By contrast, the Shannon diversity displayed a trend that appeared to be independent of DSS treatment and duration (Figure 5D). The relative abundance of *Muribaculaceae* in T1 and T2 significantly differed among the three groups (Figure S6). *Turicibacter* showed dose-dependent trends in its relative abundance, but the relative abundance of *Alistipes* had no significant trends across the time point and DSS concentration. These results indicated that the DiMDI has high robustness and accuracy unlike those of other microbial indicators.

## Discussion

For many decades, animal models have been essential for disease research. Notably, studies on IBD using a DSS-induced mouse model are the most popular in the field of intestinal microbiome research.^19^^,^^20^^,^^21^ However, it is challenging to guarantee high reproducibility in microbiome studies using mouse experiments because of small sample sizes and numerous batch effects such as sequencing depth and bioinformatics analytical methods.^16^^,^^17^ In the present study, we introduced a reliable DiMDI that can assess the microbial dysbiosis status in the mouse model. The DiMDI, an index with a high accuracy, was built using a total of four genera selected through biomarker selection methods, reflecting the multidimensional microbiome data. The DiMDI demonstrated strong robustness in the independent cohorts we studied with pregnant mice. By utilizing the developed DiMDI, we could perform highly reproducible analysis in future related studies, even within small individual laboratories. Furthermore, we propose a reproducible indicator for gut microbiome analysis by reassessing the ability of conventional microbial ecological indices to predict intestinal bacterial imbalance.

With the advancement of sequencing technology in the biology field, research involving large amounts of data has become increasingly prevalent. For transparency and data sharing, archives for storing sequencing data have been provided in NCBI SRA and EMBL-EBI.^22^^,^^23^ Several journals have also recommended data deposition on public archives. In the present study, we collected this extensive amount of data to establish a high-quality microbiome standard applicable in small-scale mouse experiments. However, we encountered two significant challenges while collecting the fragmented datasets. First, for some reasons, approximately 120 out of the 487 studies we examined neither shared their data nor provided adequate sample information. Second, the sequencing depth was insufficient possibly because of poor DNA extraction or sequencing performance.^24^

In practical terms, mouse experiments and sequencing can limit the resolution of experiments in small individual laboratories because of their high costs and labor-intensive nature. We set the minimum sample size to six and a minimum read count of 2,500 with reliable convergence in the rarefaction curves. Thus, with data that have been subjected to lower-level filtration, microbial dysbiosis can be identified in mouse models even with constraints on sample size and sequencing output. Moreover, we opted to focus on microbial genera with a prevalence exceeding 50% to assure broad applicability across diverse experimental settings. Although this approach may compromise specificity compared with species- or strain-level analysis, it carries the advantage of maintaining relevance across a wide range of experimental conditions.

Microbiome data are noisy, sparse (zero inflated), and high dimensional; thus, statistical errors more likely occur in studies with small sample sizes than in studies with large sample sizes. To mitigate these issues, we applied four different feature selection methods. First, we used the MaAsLin2, a method that can handle sparse data using clr-transformation and consider multivariates.^25^ Second, random forest modeling was used to consider outliers by building multiple trees by using a subset data.^26^^,^^27^ Third, to handle the high-dimensional microbiome dataset, we considered multicollinearity through the L1-LASSO method.^28^ Lastly, we used ALDEx2, which was developed on the basis of the Dirichlet-multinomial model, a model that enables significant feature selection even from the limited sample sizes.^29^ By opting for analytical methods that consider the dataset intricately interact with meta-communities, we suggest biomarkers with high reproducibility, even in independent studies.

In the present study, the biomarkers selected for constructing DiMDI were *Muribaculaceae*, *Alistipes*, *Bacteroides*, and *Turicibacter*. *Muribaculaceae* and *Alistipes* had high abundances in the HC group. *Muribaculaceae* and *Turicibacter* are murine-specific genera, whereas *Alistipes* and *Bacteroides* are found in the human and murine gut. The selection of murine-specific biomarkers suggests that the DiMDI can only be utilized for the DSS-induced mouse model. The lack of continuity between studies using mouse models and humans is due to the inevitable differences between them.^30^^,^^31^ For example, laboratory mice are fed a chow diet composed of plant materials, which differs from the daily diet of humans. In addition, the mouse intestine differs significantly from the human intestine in physiology and anatomy, thus mouse models cannot fully represent humans.^32^ Nevertheless, the selected genera showed consistent results with the previous studies using the mouse model.

Several studies have reported that *Muribaculaceae* is negatively correlated with colitis.^33^ *Muribaculaceae* has a strong correlation with propionate, which inhibits CD8^+^ T cell activation to tolerate immune responses.^34^^,^^35^ Thus, an increase in *Muribaculaceae* abundance may help maintain gut homeostasis*.* Furthermore, *Alistipes finegoldii* is known as a protective species against colitis. Dziarski et al.^36^ showed that treatment with *A. finegoldii* relieves colitis symptoms in mice induced with *Bacteroides eggerthii*, a species known to exacerbate colitis. Consistent with this finding, our result confirmed an increase in the relative abundance of *Bacteroides* in the gut microbiota of the DSS-induced colitis model. *Turicibacter*, which also showed high abundance in other colitis mouse models, including those treated with DSS or antibiotics,^33^^,^^37^^,^^38^ has yet to have its role in colitis fully understood. Therefore, on the basis of the four genera revealed by using large-high quality data, we anticipate that understanding of colitis can be enhanced by revealing their interactions with the host and developing treatments that target these biomarkers. In addition, DiMDI using these four genera shown high reproducibility in the independent study, so that DiMDI using four genera can be broadly used in studies using the DSS-induced mouse models without new feature selection.

Assessing gut microbial dysbiosis based on individual biomarkers identified by feature selection may compromise reproducibility because it does not reflect the high-dimensional characteristics of microbial networks. The gut microbiota is an intricate microbial ecological community,^39^ so that the colitis microbial dysbiosis may not be represented by the four selected biomarkers. Therefore, it is necessary to investigate the interactions between the four biomarkers, other gut microbes, and the host. Alternatively, predicting colitis using DiMDI proved more reliable than using individual biomarkers alone. Similarly, diversity indices, such as Shannon diversity and Chao1 index, had lower differentiation abilities than the constructed DiMDI. Moreover, the Firmicutes/Bacteroidota ratio showed the lowest accuracy for predicting colitis status in the mouse model. Several studies have reported that the Firmicutes/Bacteroidota ratio may not be representative of the gut microbial dysbiosis due to inconsistent results across studies.^40^^,^^41^^,^^42^ Microbial ecological indicators, such as Shannon diversity and Chao1 index, can be influenced by the intensity of sampling, including sequencing depth.^43^^,^^44^ Therefore, these indicators may limit because they can only be interpreted as relative comparisons between case-controls within a single cohort. Our results also revealed differences in the indices between studies even though they belong to the same HC group. Moreover, these results were aligned with our independent study that used DSS-induced pregnant mice. We observed that the DiMDI values changed in a dose-dependent manner, and the microbiome reverted to the healthy structure during the recovery phase. Therefore, these results highlighted the necessity for a reevaluation of the scales used for assessing microbial dysbiosis.

In conclusion, we extensively analyzed the gut microbiome in the DSS-induced mouse model, which is widely used in colitis-microbiome studies, to avoid statistical and analytical errors that can occur in small-scale mouse experiments. Our high-quality analysis revealed significant microbial biomarkers for determining DSS-induced microbial dysbiosis. Utilizing our developed DiMDI, we could achieve highly reproducible interpretations in future related studies even within small individual laboratories. Furthermore, we propose a new indicator for gut microbiome analyses by re-evaluating the dysbiosis prediction performance of conventional microbial ecological indicators.

### Limitations of the study

This study has several limitations. Fewer samples than those of meta-analysis in human microbiome studies were used. Despite our best efforts to collect datasets, few mouse models were used in individual studies, and some samples had undergone diet interventions or treatments, which limited our data compilation. In this study, the 16S amplicon sequencing data were applied. Given the increase in the number of studies using shotgun metagenome sequencing over the past 5 years, tracking the functions of novel biomarkers has become possible through analysis on a species or strain level. Undoubtedly, such detailed analysis is crucial to understanding their interactions with bacterial hosts. However, instead of identifying novel bacteria, we aimed to provide an accurate dysbiosis baseline for enhancing its application in colitis alleviation studies, which still predominantly use amplicon sequencing data. Lastly, a pregnant mouse model was used for validation in our independent cohort study, thereby posing another limitation. Although validation using mouse models without any environmental or biological changes is ideal, the same level of reproducibility was achieved with the pregnant mouse model. For continuity in gut microbiome studies between human and animal models, shotgun metagenome sequencing data should be collected from animal models in future studies.

## STAR★Methods

### Key resources table

**Table:** 

REAGENT or RESOURCE	SOURCE	IDENTIFIER
**Deposited data**		

Study 1; Pan et al., 2020^45^	SRA	PRJNA562994
Study 2; Gu et al., 2020^46^	SRA	PRJNA588979
Study 3; Chang et al., 2021^47^	SRA	PRJN589474
Study 4; Zhou et al., 2020^48^	SRA	PRJNA607160
Study 5; Wu et al., 2021^49^	SRA	PRJNA613584
Study 6; Chen et al., 2020^50^	SRA	PRJNA637805
Study 7; Zhou et al., 2021^51^	SRA	PRJNA663139
Study 8; Liu et al., 2021^52^	SRA	PRJNA682001
Study 9; Wu et al., 2022^53^	SRA	PRJNA765454
Study 10; Islam et al., 2022^54^	SRA	PRJNA772551
Study 11; Wang et al., 2022^55^	SRA	PRJNA779815
Study 12; Hong et al., 2022^56^	SRA	PRJNA824237
Study 13; Wang et al., 2022^57^	SRA	PRJNA845074
Study 14; Wu et al., 2019^58^	SRA	PRJNA542407
Study 15; Bai et al., 2019^59^	SRA	PRJNA563762
Study 16; Li et al., 2020^60^	SRA	PRJNA598332
Study 17; Pi et al., 2022^61^	SRA	PRJNA694703
Study 18; Wang et al., 2021^62^	SRA	PRJNA706144
Study 19; Xu et al., 2021^63^	SRA	PRJNA738354
Study 20; Vicentini et al., 2022^64^	SRA	PRJNA755589
Study 21; Wang et al., 2021^65^	SRA	PRJNA760298
Study 22; Yang et al., 2022^66^	SRA	PRJNA855543
Study 23; Wu et al., 2023^67^	SRA	PRJNA865924
This study	SRA	PRJNA1012752

**Experimental models: Organisms/strains**		

C57BL/6 J mice	DooYeol Biotech	N/A

**Oligonucleotides**		

Primer: 16S rRNA V4 region forward: 5′-CGCTCTTCCGATCTGTGNCAGCMGCCGCGGTRA-3′	This study	N/A
Primer: 16S rRNA V5 region reverse: 5′-GTGCTCTTCCGATCCGYCWATTYHTTTRAGTTT-3′	This study	N/A

**Software and algorithms**		

QIIME 2 2023.05	Bolyen et al., 2019^68^	https://docs.qiime2.org/2023.05/
Deblur2	Amir et al., 2017^69^	
SILVA database	Quast et al., 2013^70^	
R studio v4.3.1	CRAN	https://r-project.org
DiMDI (DSS-induced Microbial Dysbiosis Index)	This study	https://github.com/mjkim-micro/dimdi

### Resource availability

#### Lead contact

Further information and requests for resources and reagents should be directed to and will be fulfilled by the lead contact, Jae-Ho Shin (jhshin@knu.ac.kr).

#### Materials availability

This study did not generate new unique reagents.

#### Data and code availability

• The raw sequencing data accession IDs of all publicly available fecal microbiota samples (and their corresponding studies) used in this study are available in key resources table.
• Illumina Miseq sequences for the datasets used for DiMDI validation during the current study were publicly available as of the date of publication. Accession number is listed in the key resources table.
• R codes for calculating DiMDI in the mouse model are available from Github (https://github.com/mjkim-micro/dimdi).

### Experimental model and subject details

#### Data acquisition and quality control

The amplicon sequencing data of DSS-induced colitis mouse feces were collected through PubMed and Google Scholar for published studies by using several keywords (e.g., “DSS,” “dextran sulfate sodium,” “gut microbiome,” “gut microbiota,” “colitis mouse model”). In studies involving multiple samples taken per individual across different time points, the baseline and end-point samples were included during the period of DSS treatment in the original study. Studies pertaining to diet or medication interventions, DSS intervention with solvents (e.g., DMSO), or those with fewer than 10 samples were excluded. Samples from mice that were <4 weeks of age were also excluded from our analysis. Raw sequence files (.fastq) were downloaded from the NCBI Sequence Read Archive for the study analysis.

#### Animal and housing

All the mice were kept in conditions that were identical with respect to their environment, food, light, and temperature. All animal experimental procedures were approved by the Institutional Animal Care and Use Committee of Kyungpook National University (IACUC No., KNU-2021-0133). The ARRIVE1 reporting guidelines were implemented, and pregnant female C57BL/6 J mice were used for this study.

The mice were housed in a specific pathogen-free environment with a 12-hour light/dark cycle, 45%–65% relative humidity, and 20°C–24°C ambient temperature in open or individually ventilated cages with wood shavings for bedding and nesting materials in groups of ≤6 animals. The mice had *ad libitum* access to tap water and standard rodent chow (2018 Teklad Certified Irradiated Global 18% protein [Envigo, Indianapolis, IN, USA]). The pregnant female mice were induced with 1% and 1.5% DSS for 5 days in the second trimester of pregnancy (Figure 5A). Fecal samples were collected before (T0), during (T1), and after (T2) DSS treatment during pregnancy, and 1 (T3) and 2 weeks (T4) after postnatal recovery.

### Method details

#### DNA extraction and sequencing

Fecal microbial DNA was extracted from each sample with the QIAamp PowerFecal Pro Kit (Qiagen, Germany) following the manufacturer’s instructions. The quantity of the extracted DNA was measured using Qubit Flex Fluorometer (Thermo Fisher Scientific, USA). The 16S rRNA gene of the samples was amplified at the V4-V5 hypervariable region by using the 515F (5′-barcoded-CGCTCTTCCGATCTGTGNCAGCMGCCGCGGTRA-3′) forward primer attached to a 5′ Illumina adapter and the 907R (5′-barcode-GTGCTCTTCCGATCCGYCWATTYHTTTRAGTTT-3′) indexed reverse primer. Amplicon sizes and concentrations were measured using an Agilent 2100 bioanalyzer (Agilent Technologies, USA) and Qubit Flex Fluorometer (Thermo Fisher Scientific, USA), respectively. Each amplified 16S rRNA gene library was diluted and pooled together. Sequencing was performed using the Illumina Miseq platform with MiSeq Reagent Kit v2 (300-cycle kits) at the KNU NGS Core Facility (Daegu, South Korea).

#### Data processing

Raw reads were processed using Quantitative Insights into Microbial Ecology 2 (QIIME2; ver. 2023.5) software.^68^ Quality check, trimming, and denoising were conducted using Deblur.^69^ The sequences with low quality (<Q30) and read counts (<2,500) were excluded. Amplicon sequence variants (ASVs) were identified microbial taxonomic levels by using the SILVA database and the naïve Bayes pre-trained QIIME2 classifier.^70^ Mitochondrial, chloroplast, and unclassified/unassigned sequences were removed from the assessed ASVs. Studies with fewer than six samples were excluded after they were processed. All sequencing data were separately processed for each study; statistical analysis was conducted using ‘qiime feature-table merge’ command.

#### Construction of the DiMDI model

Microbial biomarkers were identified using genera in the mouse fecal microbiota. First, unclassified or unknown genera were excluded. Rare genera with low abundance (<0.1%) or prevalence (<50%) were filtered (196 genera) to increase the reproducibility of the model. Then, four different statistical analyses (ALDEx2, random forest, MaAsLin2, and L1-regularized [LASSO] logistic regression) were performed to discover the microbial biomarkers.^25^^,^^26^^,^^28^^,^^29^^,^^43^ The feature selections using ALDEx2, MaAsLin2, and L1-regularized (LASSO) logistic regression were performed with default parameters. In particular, the significant features were selected based on Benjamini-Hochberg corrected p-value for ALDEx2 and MaAsLin2. MaAsLin2 further evaluated the significant features by using linear regression model. In the L1-LASSO logistic regression, the significant features were selected using a regularization parameter that constructs the most accurate model. For feature selection using random forest, the entire sample set was randomly divided into training and test sets. The training performance was evaluated by 5-run 10-fold cross-validation, with the automatic execution of hyperparameter tuning. An initial model was constructed to calculate importance based on the mean decrease in Gini score, indicating the purity of classification splits for given variables, by using all bacterial genus variables. Features were incrementally added to the model based on their importance, and the best final model was selected by calculating the area under curve (AUC) of the receiver operating characteristic (ROC) curve. Among the genera chosen by four feature selection methods, DiMDI was constructed using the genera that were chosen by three or more methods concurrently. The DiMDI was calculated by the modified equation based on the previous study:^5^

$$\text{DiMDI}=\log\left\{1+\frac{\sum_{i=n}Relative\;abudnance\left(DSS\;colitis\;enriched\right)i}{\left(1+\sum_{j=m}Relative\;abundance\left(Healthy\;enriched\right)j\right)}\right\}$$

where Relativeabudnance(DSScolitisenriched)i is the relative abundance of genera enriched in DSS colitis feces, Relativeabundance(Healthyenriched)j is the relative abundance of genera enriched in healthy feces, and the constant 1 is added to avoid zero values. R scripts for calculating DiMDI in the mouse model are available at GitHub (https://github.com/mjkim-micro).

### Quantification and statistical analysis

Statistical analyses and visualizations were conducted in the R studio (v4.3.1; http://www.r-project.org/). The principal coordinate analysis (PCoA) based on Bray–Curtis dissimilarity distance matrix was performed using vegan (v2.6-4) and phyloseq (v1.44.0) R packages. Comparisons between the HC and UH groups were performed using a T-test. The correlation between the concentration of DSS induction and alpha indices (Shannon and Chao1) was measured by Pearson’s correlation coefficient and visualized by ggpubr (v0.6.0). Microbial biomarkers were selected using randomForest (v4.7-1.1), caret (v6.0-93), SIAMCAT (v2.2.0), ALDEx2 (v1.32.0), and MaAsLin2 (v1.14.1) R packages.^25^^,^^28^^,^^29^^,^^71^ The ROC curve was constructed to evaluate the performance of the DiMDI and each marker. The performances in different models were compared using DeLong’s test. These performance evaluations were conducted using the multipleROC (v0.1.0) R package. Wilcoxon signed rank test was used to compare the HC and UH groups within each study. The three groups in the independent cohort were compared using the Kruskal–Wallis test, and post-hoc comparisons were performed using Dunn’s test. The confusion matrix was generated by the caret R package.

## References

[1] Cani P.D. (2018). Human gut microbiome: hopes, threats and promises. Gut.

[2] Shreiner A.B., Kao J.Y., Young V.B. (2015). The gut microbiome in health and in disease. Curr. Opin. Gastroenterol..

[3] Lavelle A., Hill C. (2019). Gut Microbiome in Health and Disease: Emerging Diagnostic Opportunities. Gastroenterol. Clin. North Am..

[4] Schlaberg R. (2020). Microbiome Diagnostics. Clin. Chem..

[5] Gevers D., Kugathasan S., Denson L.A., Vazquez-Baeza Y., Van Treuren W., Ren B., Schwager E., Knights D., Song S.J., Yassour M. (2014). The treatment-naive microbiome in new-onset Crohn's disease. Cell Host Microbe.

[6] Jo Y.J., Tagele S.B., Pham H.Q., Jung Y., Ibal J.C., Choi S., Kang G.U., Park S., Kang Y., Kim S. (2020). In Situ Profiling of the Three Dominant Phyla Within the Human Gut Using TaqMan PCR for Pre-Hospital Diagnosis of Gut Dysbiosis. Int. J. Mol. Sci..

[7] Luo Y., Cui X., Cheruba E., Chua Y.K., Ng C., Tan R.Z., Tan K.K., Cheow L.F. (2022). SAMBA: A Multicolor Digital Melting PCR Platform for Rapid Microbiome Profiling. Small Methods.

[8] Xia G.H., You C., Gao X.X., Zeng X.L., Zhu J.J., Xu K.Y., Tan C.H., Xu R.T., Wu Q.H., Zhou H.W. (2019). Stroke Dysbiosis Index (SDI) in Gut Microbiome Are Associated With Brain Injury and Prognosis of Stroke. Front. Neurol..

[9] Soares R.C., Camargo-Penna P.H., de Moraes V.C., De Vecchi R., Clavaud C., Breton L., Braz A.S., Paulino L.C. (2016). Dysbiotic Bacterial and Fungal Communities Not Restricted to Clinically Affected Skin Sites in Dandruff. Front. Cell. Infect. Microbiol..

[10] Yu J., Feng Q., Wong S.H., Zhang D., Liang Q.Y., Qin Y.W., Tang L.Q., Zhao H., Stenvang J., Li Y.L. (2017). Metagenomic analysis of faecal microbiome as a tool towards targeted non-invasive biomarkers for colorectal cancer. Gut.

[11] Xavier R.J., Podolsky D.K. (2007). Unravelling the pathogenesis of inflammatory bowel disease. Nature.

[12] Yu Y.R., Rodriguez J.R. (2017). Clinical presentation of Crohn's, ulcerative colitis, and indeterminate colitis: Symptoms, extraintestinal manifestations, and disease phenotypes. Semin. Pediatr. Surg..

[13] Chassaing B., Darfeuille-Michaud A. (2011). The commensal microbiota and enteropathogens in the pathogenesis of inflammatory bowel diseases. Gastroenterology.

[14] Cooper H.S., Murthy S.N., Shah R.S., Sedergran D.J. (1993). Clinicopathologic study of dextran sulfate sodium experimental murine colitis. Lab. Invest..

[15] Okayasu I., Hatakeyama S., Yamada M., Ohkusa T., Inagaki Y., Nakaya R. (1990). A novel method in the induction of reliable experimental acute and chronic ulcerative colitis in mice. Gastroenterology.

[16] Hertzog M.A. (2008). Considerations in determining sample size for pilot studies. Res. Nurs. Health.

[17] Gibbons S.M., Duvallet C., Alm E.J. (2018). Correcting for batch effects in case-control microbiome studies. PLoS Comput. Biol..

[18] Wang Y., LeCao K.A. (2020). Managing batch effects in microbiome data. Brief Bioinform..

[19] Wirtz S., Neurath M.F. (2007). Mouse models of inflammatory bowel disease. Adv. Drug Deliv. Rev..

[20] Wirtz S., Neufert C., Weigmann B., Neurath M.F. (2007). Chemically induced mouse models of intestinal inflammation. Nat. Protoc..

[21] Li M., Wu Y., Hu Y., Zhao L., Zhang C. (2018). Initial gut microbiota structure affects sensitivity to DSS-induced colitis in a mouse model. Sci. China Life Sci..

[22] Leinonen R., Sugawara H., Shumway M., International Nucleotide Sequence Database C. (2011). The sequence read archive. Nucleic Acids Res..

[23] Leinonen R., Akhtar R., Birney E., Bower L., Cerdeno-Tarraga A., Cheng Y., Cleland I., Faruque N., Goodgame N., Gibson R. (2011). The European Nucleotide Archive. Nucleic Acids Res..

[24] Bharti R., Grimm D.G. (2021). Current challenges and best-practice protocols for microbiome analysis. Brief Bioinform..

[25] Mallick H., Rahnavard A., McIver L.J., Ma S., Zhang Y., Nguyen L.H., Tickle T.L., Weingart G., Ren B., Schwager E.H. (2021). Multivariable association discovery in population-scale meta-omics studies. PLoS Comput. Biol..

[26] Dietterich T.G. (2000). An experimental comparison of three methods for constructing ensembles of decision trees: Bagging, boosting, and randomization. Mach. Learn..

[27] Breiman L. (1996). Bagging predictors. Mach. Learn..

[28] Wirbel J., Zych K., Essex M., Karcher N., Kartal E., Salazar G., Bork P., Sunagawa S., Zeller G. (2021). Microbiome meta-analysis and cross-disease comparison enabled by the SIAMCAT machine learning toolbox. Genome Biol..

[29] Fernandes A.D., Macklaim J.M., Linn T.G., Reid G., Gloor G.B. (2013). ANOVA-like differential expression (ALDEx) analysis for mixed population RNA-Seq. PLoS One.

[30] Carmody R.N., Gerber G.K., Leuvano J.M., Gatti D.M., Somes L., Svenson K.L., Turnbaugh P.J. (2015). Diet Dominates Host Genotype in Shaping the Murine Gut Microbiota. Cell Host Microbe.

[31] Nguyen T.L.A., Vieira-Silva S., Liston A., Raes J. (2015). How informative is the mouse for human gut microbiota research?. Dis. Model Mech..

[32] Treuting P.M., Dintzis S., Montine K.S. (2017).

[33] Shang L., Liu H., Yu H., Chen M., Yang T., Zeng X., Qiao S. (2021). Core Altered Microorganisms in Colitis Mouse Model: A Comprehensive Time-Point and Fecal Microbiota Transplantation Analysis. Antibiotics (Basel).

[34] Smith B.J., Miller R.A., Ericsson A.C., Harrison D.C., Strong R., Schmidt T.M. (2019). Changes in the gut microbiome and fermentation products concurrent with enhanced longevity in acarbose-treated mice. BMC Microbiol..

[35] Meehan C.J., Beiko R.G. (2014). A phylogenomic view of ecological specialization in the Lachnospiraceae, a family of digestive tract-associated bacteria. Genome Biol. Evol..

[36] Dziarski R., Park S.Y., Kashyap D.R., Dowd S.E., Gupta D. (2016). Pglyrp-Regulated Gut Microflora Prevotella falsenii, Parabacteroides distasonis and Bacteroides eggerthii Enhance and Alistipes finegoldii Attenuates Colitis in Mice. PLoS One.

[37] Lee S.M., Kim N., Nam R.H., Park J.H., Choi S.I., Park Y.T., Kim Y.R., Seok Y.J., Shin C.M., Lee D.H. (2019). Gut microbiota and butyrate level changes associated with the long-term administration of proton pump inhibitors to old rats. Sci. Rep..

[38] Bernstein C.N., Forbes J.D. (2017). Gut Microbiome in Inflammatory Bowel Disease and Other Chronic Immune-Mediated Inflammatory Diseases. Inflamm. Intest. Dis..

[39] Kosiewicz M.M., Zimheld A.L., Alard P. (2011). Gut microbiota, immunity, and disease: a complex relationship. Front. Microbiol..

[40] Finucane M.M., Sharpton T.J., Laurent T.J., Pollard K.S. (2014). A Taxonomic Signature of Obesity in the Microbiome? Getting to the Guts of the Matter. PLoS One.

[41] Walters W.A., Xu Z., Knight R. (2014). Meta-analyses of human gut microbes associated with obesity and IBD. FEBS Lett..

[42] Sze M.A., Schloss P.D. (2016). Looking for a Signal in the Noise: Revisiting Obesity and the Microbiome. mBio.

[43] Fisher R.A., Corbet A.S., Williams C.B. (1943). The relation between the number of species and the number of individuals in a random sample of an animal population. J. Anim. Ecol..

[44] Willis A.D. (2019). Rarefaction, Alpha Diversity, and Statistics. Front. Microbiol..

[45] Pan G., Liu B., Han M., Gao L., Xu G., Du Q., Xie L. (2020). Kuijieling, a Chinese medicine alleviates DSS-induced colitis in C57BL/6J mouse by improving the diversity and function of gut microbiota. FEMS Microbiol. Lett..

[46] Gu Z., Zhu Y., Jiang S., Xia G., Zhang X., Zhang J., Shen X. (2020). Tilapia head glycolipids reduce inflammation by regulating the gut microbiota in dextran sulphate sodium-induced colitis mice. Food Funct..

[47] Chang C.S., Liao Y.C., Huang C.T., Lin C.M., Cheung C.H.Y., Ruan J.W., Yu W.H., Tsai Y.T., Lin I.J., Huang C.H. (2021). Identification of a gut microbiota member that ameliorates DSS-induced colitis in intestinal barrier enhanced Dusp6-deficient mice. Cell Rep..

[48] Zhou B.G., Liu F.C., Zhao H.M., Zhang X.Y., Wang H.Y., Liu D.Y. (2020). Regulatory effect of Zuojin Pill on correlation with gut microbiota and Treg cells in DSS-induced colitis. J. Ethnopharmacol..

[49] Wu Z., Huang S., Li T., Li N., Han D., Zhang B., Xu Z.Z., Zhang S., Pang J., Wang S. (2021). Gut microbiota from green tea polyphenol-dosed mice improves intestinal epithelial homeostasis and ameliorates experimental colitis. Microbiome.

[50] Chen F., Yin Y.T., Zhao H.M., Wang H.Y., Zhong Y.B., Long J., Liu D.Y. (2020). Sishen pill treatment of DSS-induced colitis via regulating interaction with inflammatory dendritic cells and gut microbiota. Front. Physiol..

[51] Zhou H., Zeng X., Sun D., Chen Z., Chen W., Fan L., Limpanont Y., Dekumyoy P., Maleewong W., Lv Z. (2021). Monosexual cercariae of schistosoma japonicum infection protects against DSS-induced colitis by shifting the Th1/Th2 balance and modulating the gut microbiota. Front. Microbiol..

[52] Liu Z., Liao W., Zhang Z., Sun R., Luo Y., Chen Q., Li X., Lu R., Ying Y. (2021). Metformin affects gut microbiota composition and diversity associated with amelioration of dextran sulfate sodium-induced colitis in mice. Front. Pharmacol..

[53] Wu Z., Liu X., Huang S., Li T., Zhang X., Pang J., Zhao J., Chen L., Zhang B., Wang J. (2022). Milk fat globule membrane attenuates acute colitis and secondary liver injury by improving the mucus barrier and regulating the gut microbiota. Front. Immunol..

[54] Islam S.S., Ryu H.M., Sohn S. (2022). Tetragenococcus halophilus alleviates intestinal inflammation in mice by altering gut microbiota and regulating dendritic cell activation via CD83. Cells.

[55] Wang H., Huang J., Ding Y., Zhou J., Gao G., Han H., Zhou J., Ke L., Rao P., Chen T. (2022). Nanoparticles isolated from porcine bone soup ameliorated dextran sulfate sodium-induced colitis and regulated gut microbiota in mice. Front. Nutr..

[56] Hong Z.S., Xie J., Wang X.F., Dai J.J., Mao J.Y., Bai Y.Y., Sheng J., Tian Y. (2022). Moringa oleifera Lam. peptide remodels intestinal mucosal barrier by inhibiting JAK-STAT activation and modulating gut microbiota in colitis. Front. Immunol..

[57] Wang W., Xu Y., Wang X., Chu Y., Zhang H., Zhou L., Zhu H., Li J., Kuai R., Zhou F. (2022). Swimming Impedes Intestinal Microbiota and Lipid Metabolites of Tumorigenesis in Colitis-Associated Cancer. Front. Oncol..

[58] Wu M., Li P., An Y., Ren J., Yan D., Cui J., Li D., Li M., Wang M., Zhong G. (2019). Phloretin ameliorates dextran sulfate sodium-induced ulcerative colitis in mice by regulating the gut microbiota. Pharmacol. Res..

[59] Bai Y., Wang S., Xu W., Weng Y., Zhu S., Sheng H., Zhu J., Zhang F. (2019). Cinobufacini ameliorates experimental colitis via modulating the composition of gut microbiota. PLoS One.

[60] Li P., Xiao N., Zeng L., Xiao J., Huang J., Xu Y., Chen Y., Ren Y., Du B. (2020). Structural characteristics of a mannoglucan isolated from Chinese yam and its treatment effects against gut microbiota dysbiosis and DSS-induced colitis in mice. Carbohydr. Polym..

[61] Pi Y., Zhang X., Wu Y., Wang Z., Bai Y., Liu X., Han D., Zhao J., Tobin I., Zhao J. (2022). Alginate alleviates dextran sulfate sodium-induced colitis by promoting Bifidobacterium animalis and intestinal hyodeoxycholic acid synthesis in mice. Microbiol. Spectr..

[62] Wang C., Li S., Hong K., Yu L., Tian F., Zhao J., Zhang H., Chen W., Zhai Q. (2021). The roles of different Bacteroides fragilis strains in protecting against DSS-induced ulcerative colitis and related functional genes. Food Funct..

[63] Xu H.M., Huang H.L., Liu Y.D., Zhu J.Q., Zhou Y.L., Chen H.T., Xu J., Zhao H.L., Guo X., Shi W. (2021). Selection strategy of dextran sulfate sodium-induced acute or chronic colitis mouse models based on gut microbial profile. BMC Microbiol..

[64] Vicentini F.A., Szamosi J.C., Rossi L., Griffin L., Nieves K., Bihan D., Lewis I.A., Pittman Q.J., Swain M.G., Surette M.G. (2022). Colitis-associated microbiota drives changes in behaviour in male mice in the absence of inflammation. Brain Behav. Immun..

[65] Wang Y., Zhang J., Xu L., Ma J., Lu M., Ma J., Lu M., Ma J., Liu Z., Wang F., Tang X. (2021). Modified gegen qinlian decoction regulates Treg/Th17 balance to ameliorate DSS-induced acute experimental colitis in mice by altering the gut microbiota. Front. Pharmacol..

[66] Yang R., Shan S., An N., Liu F., Cui K., Shi J., Li H., Li Z. (2022). Polyphenols from foxtail millet bran ameliorate DSS-induced colitis by remodeling gut microbiome. Front. Nutr..

[67] Wu Y., Ran L., Yang Y., Gao X., Peng M., Liu S., Sun L., Wan J., Wang Y., Yang K. (2023). Deferasirox alleviates DSS-induced ulcerative colitis in mice by inhibiting ferroptosis and improving intestinal microbiota. Life Sci..

[68] Bolyen E., Rideout J.R., Dillon M.R., Bokulich N.A., Abnet C.C., Al-Ghalith G.A., Alexander H., Alm E.J., Arumugam M., Asnicar F. (2019). Reproducible, interactive, scalable and extensible microbiome data science using QIIME 2. Nat. Biotechnol..

[69] Amir A., McDonald D., Navas-Molina J.A., Kopylova E., Morton J.T., Zech Xu Z., Kightley E.P., Thompson L.R., Hyde E.R., Gonzalez A., Knight R. (2017). Deblur rapidly resolves single-nucleotide community sequence patterns. mSystems.

[70] Quast C., Pruesse E., Yilmaz P., Gerken J., Schweer T., Yarza P., Peplies J., Glockner F.O. (2013). The SILVA ribosomal RNA gene database project: improved data processing and web-based tools. Nucleic Acids Res..

[71] Kuhn M. (2008). Building Predictive Models in R Using the caret Package. J. Stat. Softw..

