# Supplementary material for: Microbial dysbiosis index for assessing colitis status in mouse models: A systematic review and meta-analysis
Source: iScience. 2023 Dec 6;27(1):108657. doi: 10.1016/j.isci.2023.108657 (PMC10777064; doi:10.1016/j.isci.2023.108657)
Supplement: Document S1. Figures S1–S6 and Table S1 [file mmc1.pdf]

iScience, Volume *27*

Supplemental information

Microbial dysbiosis index for assessing colitis status in mouse models: A systematic review and meta-analysis

Min-Ji Kim, Da-Ryung Jung, Ji-Min Lee, Ikwhan Kim, HyunWoo Son, Eun Soo Kim, and Jae-Ho Shin

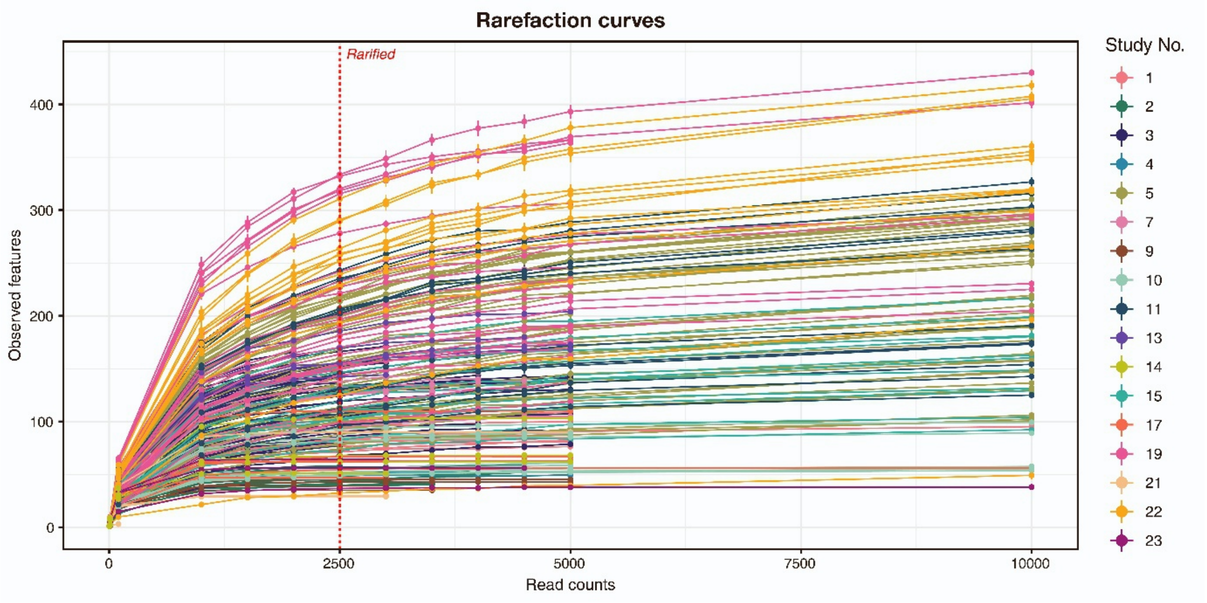

Figure S1. A total of 117 samples with low read counts were excluded and the remaining samples were rarefied at 2,500, related to Figure 1. The red dashed line indicates the rarified read count for downstream analysis.

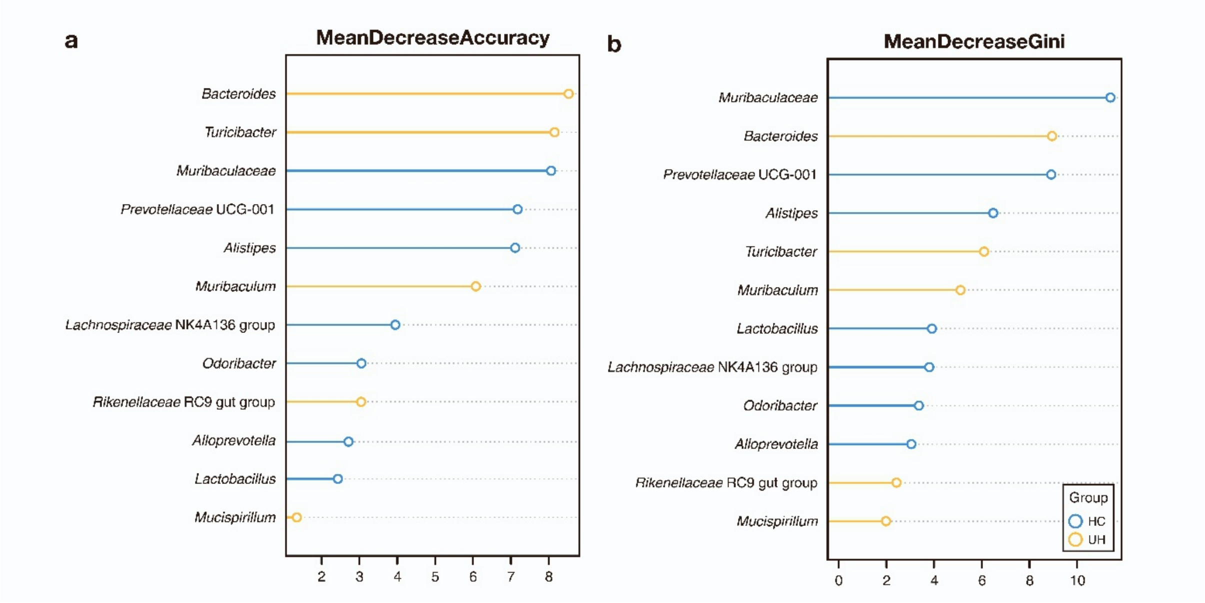

Figure S2. The random forest model was constructed based on the feature importance indicated by MeanDecreaseAccuracy and MeanDecreaseGini, related to Figure 3. Genus importance determined by the random forest modeling using the mean decrease in **(a)** MeanDecreaseAccuracy and **(b)** MeanDecreaseGini.

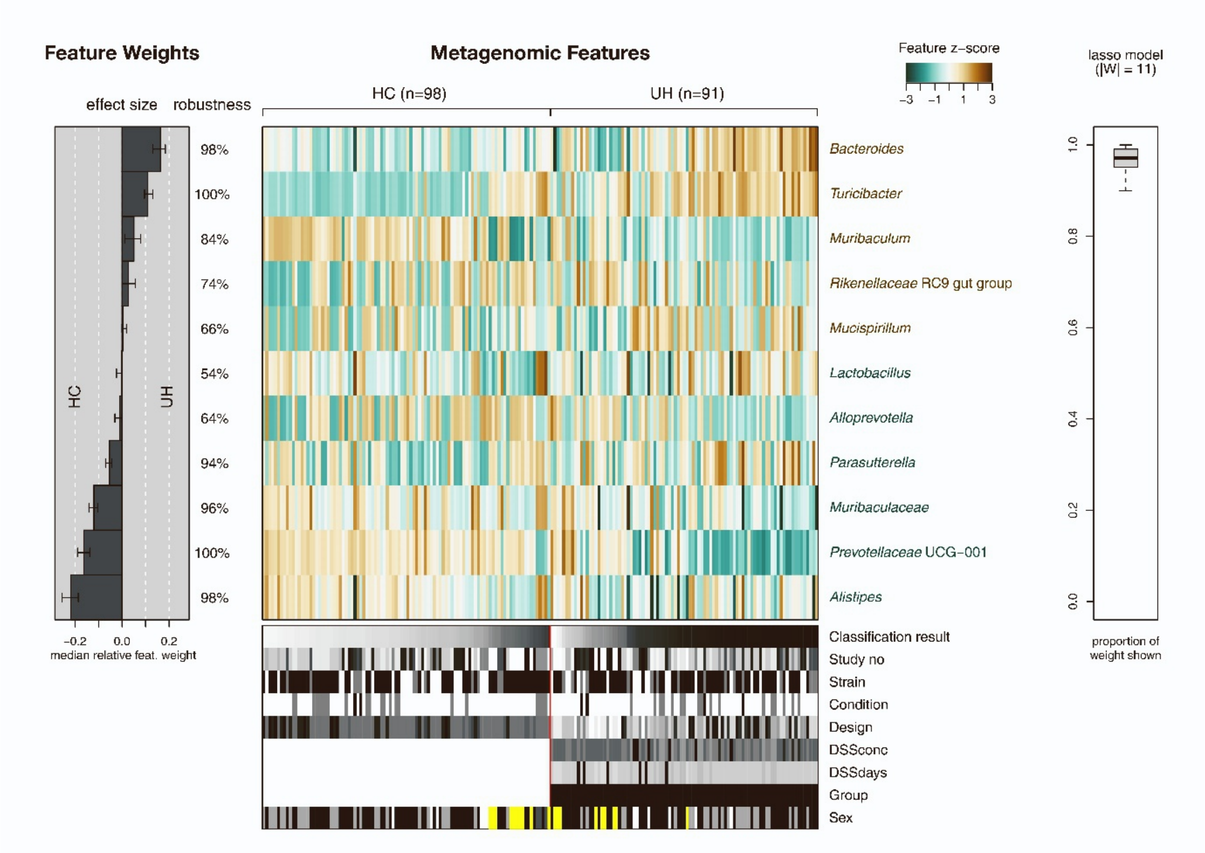

Figure S3. The selected four biomarkers also showed a high value of effect size in the L1-LASSO logistic model, related to Figure 3. Feature importance for each genus included in the majority of L1-LASSO logistic models fitted during cross validation. A heatmap displays the normalized values across all samples, along with the classification result and user-defined meta-variables (bottom).

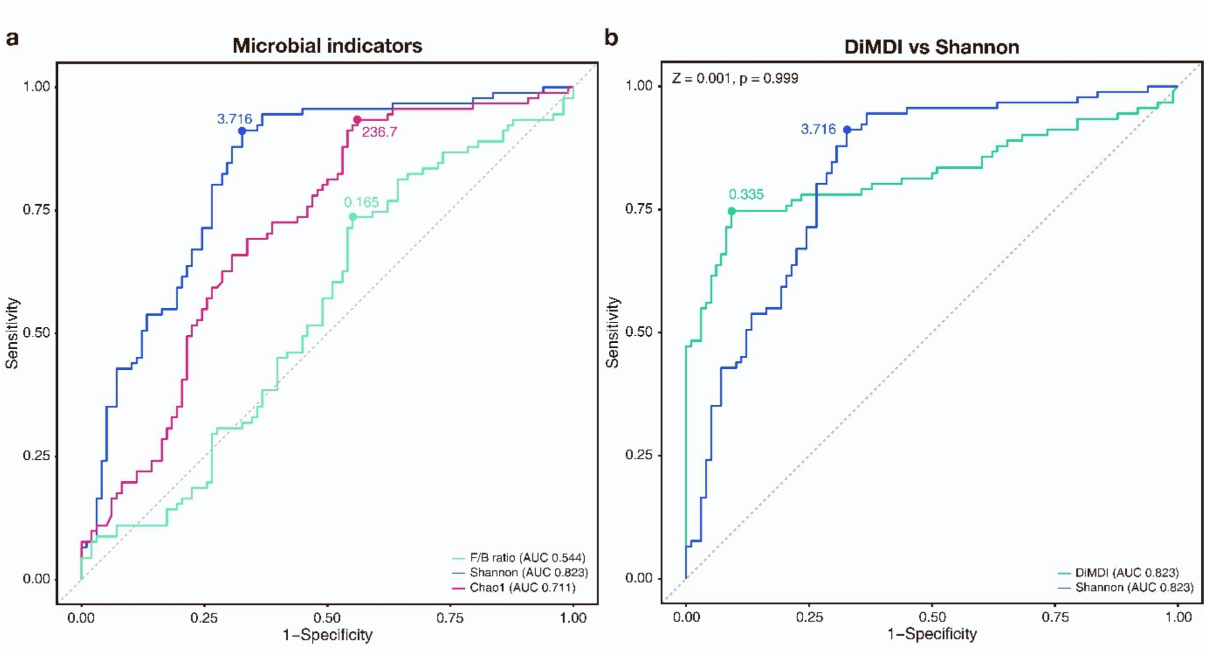

Figure S4. The DiMDI showed the highest accuracy compared to other microbial ecological indicators, such as Shannon index and F/B ratio, related to Figure 4. **(a)** Area under the curve (AUC)-receiver operating curve (ROC) of microbial indicators to classify UH from HC. **(b)** Comparison of area under the curves (AUCs) for DiMDI and Shannon. AUCs between two indices were compared using DeLong's test.

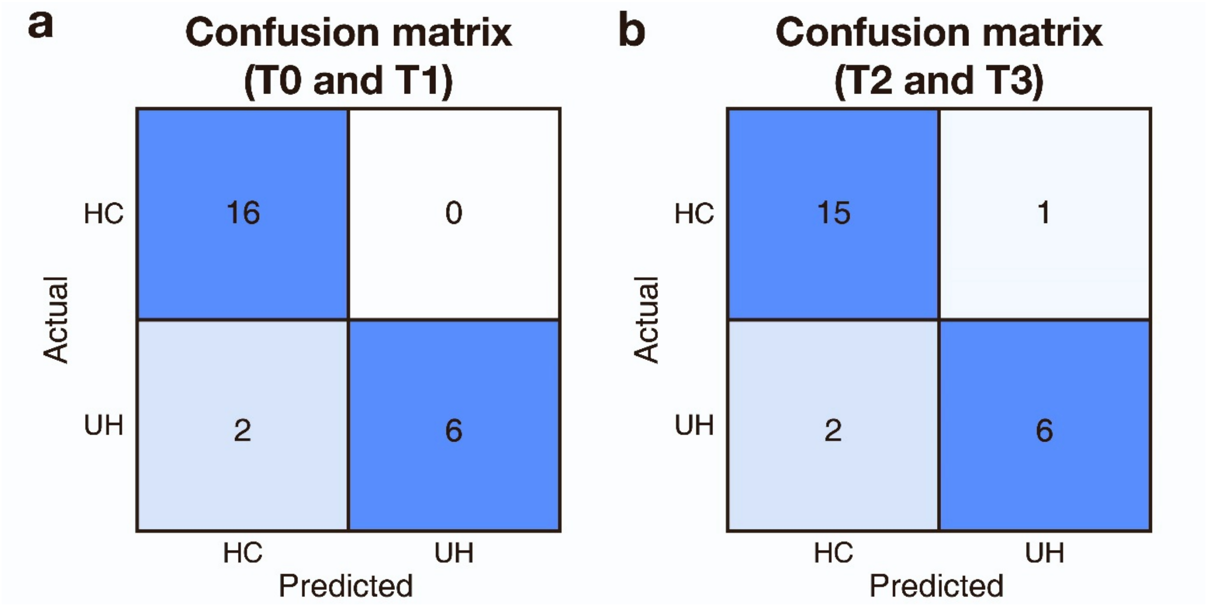

Figure S5. The DiMDI had high reproducibility in the independent study, related to Figure 5. The confusion matrix of the DiMDI applied to **(a)** T0 and T1, and **(b)** T2 and T3 in the independent cohort.

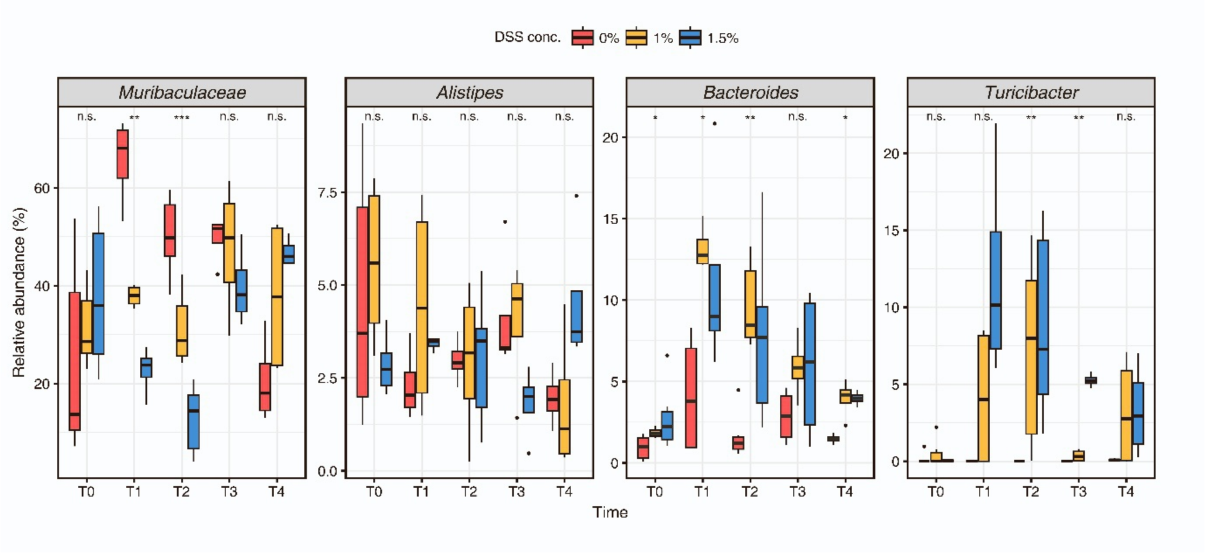

Figure S6. The relative abundances of the selected microbial biomarkers showed a consistent trend with the meta-analysis results across time points and DSS concentrations, related to Figure 3 and Figure 5. Kruskal–Wallis test was used to compare three groups within the time point. Significances were depicted by n.s. (non-significant; >0.05), * (<0.05), ** (<0.01), and *** (<0.001).

Table S1. A total of four genera were selected as important features as overlapping in four different feature selection methods, related to Figure 3.

Features	Feature selection methods	Relative abundance (mean ± sd, %)		*P* value [a]
		HC	UH	
Alistipes	Random forest, LASSO regression, ALDEx2, MaAsLin2	7.004 ± 5.370	2.471 ± 2.920	**< 0.001**
Alloprevotella	Random forest, LASSO regression	4.039 ± 6.450	1.862 ± 3.862	**0.005**
Bacteroides	Random forest, LASSO regression, ALDEx2, MaAsLin2	6.600 ± 11.22	23.32 ± 21.87	**< 0.001**
Desulfovibrio	LASSO regression	0.900 ± 1.711	0.505 ± 1.084	0.058
Lachnospiraceae NK4A136 group	Random forest	3.636 ± 5.447	1.548 ± 2.503	**< 0.001**
Lactobacillus	Random forest, LASSO regression	7.851 ± 14.46	5.298 ± 9.926	0.156
Mucispirillum	Random forest, LASSO regression	1.002 ± 2.445	1.276 ± 4.340	0.597
Muribaculaceae	Random forest, LASSO, ALDEx2, MaAsLin2	39.03 ± 18.33	17.17 ± 20.45	**< 0.001**
Muribaculum	Random forest, LASSO regression	0.744 ± 0.872	0.196 ± 0.401	**< 0.001**
Odoribacter	Random forest, MaAsLin2	6.436 ± 8.106	3.982 ± 6.467	**0.022**
Parasutterella	LASSO regression	0.187 ± 0.345	0.383 ± 0.916	0.057
Prevotellaceae UCG-001	Random forest, LASSO regression	2.100 ± 2.242	1.102 ± 2.950	**0.010**
Rikenellaceae RC9 gut group	Random forest, LASSO regression	1.023 ± 2.486	1.050 ± 3.552	0.953
Turicibacter	Random forest, LASSO, ALDEx2	0.328 ± 0.842	3.163 ± 8.579	**0.002**

[a] T-test was applied to compare the HC and UH groups, with significant values highlighted in bold.
